# Lisdexamfetamine in combination with guanfacine as an effective treatment in the management of behavioral disturbances in patients with Attention Deficit Hyperactivity Disorder (ADHD) and Autism Spectrum Disorder (ASD). Case report

**DOI:** 10.1192/j.eurpsy.2023.1554

**Published:** 2023-07-19

**Authors:** P. Del Sol Calderon, A. Izquierdo de la Puente, M. García Moreno, R. Fernández Fernandez

**Affiliations:** 1 Psychiatry, Hospital Puerta de Hierro, Majadahonda; 2 Psychiatry, Hospital Infanta Cristina, Madrid, Spain

## Abstract

**Introduction:**

We often find it challenging to manage hyperactivity, low frustration tolerance and angry outbursts in patients with ASD and comorbid ADHD. Fewer drugs are approved for these disorders and these patients are more likely to develop adverse effects.

**Objectives:**

The aim of this case is to show how the combination of lisdexamfetamine together with guanfacine has very positive effects on anger outbursts and boundary heteroaggressiveness in patients with ASD and ADHD.

**Methods:**

Case report and literature review

**Results:**

This is a 14-year-old minor admitted to the psychiatric unit after physical aggression against his family due to anger after removal of video games, requiring police intervention. He has been diagnosed since he was 11 years old with ADHD and Autism Spectrum Disorder. He was being treated with methylphenidate 54 mg and aripiprazole 10 mg. Since the beginning of the admission, the following pharmacological adjustment has been made: Methylphenidate is substituted by lisdexamfetamine up to 50 mg per day. Guanfacine has been started up to 4 mg per day and the dose of aripiprazole has been maintained. The patient had no adverse effects with adequate tolerance without sedation, hyporexia or hypotension. With this adjustment, improvement was found in the levels of restlessness and hyperactivity. The patient expressed a subjective improvement in the levels of restlessness and with a notable improvement in attention in the hospital classroom. An improvement in emotional regulation was also observed, with more tolerance to the imposition of limits, without an explosion of anger in the face of any rule during admission

**Conclusions:**

The management of hyperactivity and episodes of low frustration tolerance in patients with ASD and ADHD is complex. Many studies point out the time-limited use of some antipsychotics such as risperidone or aripiprazole. This work aims to show guanfacine in combination with lisdexamfetamine as an excellent combination for the management of agitation and rage explosion in these patients. In addition, the profile of adverse effects at metabolic level is much better than that of atypical antipsychotics.

**Reference:**

- Extended-Release Guanfacine for Hyperactivity in Children With Autism Spectrum Disorder. Lawrence Scahill et al. Am J Psychiatry. 2015 Dec.

**Disclosure of Interest:**

None Declared

